# Extracellular Production and Degradation of Superoxide in the Coral *Stylophora pistillata* and Cultured *Symbiodinium*

**DOI:** 10.1371/journal.pone.0012508

**Published:** 2010-09-14

**Authors:** Eldad Saragosti, Dan Tchernov, Adi Katsir, Yeala Shaked

**Affiliations:** 1 Interuniversity Institute for Marine Sciences, Eilat, Israel; 2 Department of Evolution, Systematics and Ecology, Alexander Silberman Institute of Life Sciences, Hebrew University of Jerusalem, Eilat, Israel; 3 Department of Life Sciences, Ben Gurion University of the Negev, Beer Sheva, Israel; 4 Fredy & Nadine Herrmann Institute of Earth Sciences, Hebrew University of Jerusalem, Eilat, Israel; Northern Fisheries Centre, Australia

## Abstract

**Background:**

Reactive oxygen species (ROS) are thought to play a major role in cell death pathways and bleaching in scleractinian corals. Direct measurements of ROS in corals are conspicuously in short supply, partly due to inherent problems with ROS quantification in cellular systems.

**Methodology/Principal Findings:**

In this study we characterized the dynamics of the reactive oxygen species superoxide anion radical (O_2_^−^) in the external *milieu* of the coral *Stylophora pistillata*. Using a sensitive, rapid and selective chemiluminesence-based technique, we measured extracellular superoxide production and detoxification activity of symbiont (non-bleached) and aposymbiont (bleached) corals, and of cultured *Symbiodinium* (from clades A and C). Bleached and non-bleached *Stylophora* fragments were found to produce superoxide at comparable rates of 10^−11^–10^−9^ mol O_2_^−^ mg protein^−1^ min^−1^ in the dark. In the light, a two-fold enhancement in O_2_^−^ production rates was observed in non-bleached corals, but not in bleached corals. Cultured *Symbiodinium* produced superoxide in the dark at a rate of 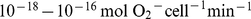. Light was found to markedly enhance O_2_^−^ production. The NADPH Oxidase inhibitor Diphenyleneiodonium chloride (DPI) strongly inhibited O_2_^−^ production by corals (and more moderately by algae), possibly suggesting an involvement of NADPH Oxidase in the process. An extracellular O_2_^−^ detoxifying activity was found for bleached and non-bleached *Stylophora* but not for *Symbiodinium*. The O_2_^−^ detoxifying activity was partially characterized and found to resemble that of the enzyme superoxide dismutase (SOD).

**Conclusions/Significance:**

The findings of substantial extracellular O_2_^−^ production as well as extracellular O_2_^−^ detoxifying activity may shed light on the chemical interactions between the symbiont and its host and between the coral and its environment. Superoxide production by *Symbiodinium* possibly implies that algal bearing corals are more susceptible to an internal build-up of O_2_^−^, which may in turn be linked to oxidative stress mediated bleaching.

## Introduction

Reactive oxygen species (ROS), consisting of the superoxide anion radical (O_2_^−^), hydrogen peroxide (H_2_O_2_), the hydroxyl radical (^•^OH) and hydroxyl radical ion (OH^−^) are formed by a variety of chemical, photochemical and biological pathways in a stepwise reduction of oxygen [1]. Superoxide (O_2_^−^), a biologically common and highly reactive oxygen species which is at the heart of this research, can react with nitric oxide (NO) to form the toxic product peroxynitrite (ONOO^−^, [2]) or dismutate to form hydrogen peroxide (H_2_O_2_) [1]. Either the combination of H_2_O_2_ with metal ions (e.g. iron) or the breakdown of ONOO^−^ can produce the highly toxic hydroxyl radical (^•^OH). To prevent such undesired reactions the intracellular levels of superoxide are tightly regulated by the enzyme superoxide dismutase (SOD) that catalyzes the dismutation of two superoxide radicals to hydrogen peroxide and oxygen [3].

ROS are common by-products of normal aerobic cell metabolism and at low levels they serve as important signalling molecules [1]. Nonetheless, their production and accumulation beyond the capacity of an organism to efficiently quench them, a state known as oxidative stress, results in extensive damage to various cellular components and eventually to cell demise [4], [5], [6]. In Cnidarians, oxidative stress has been shown to play a role in coral bleaching and apoptosis [7], [8], [9]. Many previous studies have examined the physiological and biochemical response of the coral hosts and/or symbiont algae to oxidative stress by looking at gene expression [10], protein synthesis [11], [12], antioxidant activity [13] and oxidative damage to proteins and DNA [14]. Direct *in vivo* quantification of intercellular levels of ROS was attempted in several studies using general or species-specific dyes (mostly for hydrogen peroxide [15], [16]). Despite the ease of use of fluorescent dyes in cellular systems, there are many inherent limitations to this methodology and it is highly artifact prone [17], [18], [19]. Our intensive preliminary work on intracellular ROS in *Stylophora pistillata* and its symbionts with the fluorescent dye 2′, 7′-dichlorofluorescin diacetate (H_2_DCF-DA) identified many problems with this probe. Consequently the emphasis of the present study was placed on measuring superoxide production and anti-oxidant activity in the immediate surroundings of the coral; its external *milieu*.

The experimental choice of studying the extracellular dynamics of superoxide (that is the production/release of superoxide as well as antioxidant agents/activity) holds both promises and challenges. Its main advantage is that it enables precise superoxide quantification, unlike the situation in complex cellular systems [17], [18], [19]. This is because the chemistry of the water surrounding the coral is simpler and better controlled as compared to that within the organisms. This in turn allows for easy assessment of the reactivity of O_2_^−^ that enables straight-forward corrections for its decay during measurements. The challenge in applying this method for probing oxidative stress in corals is to define whether these extracellular fluxes reflect intracellular processes. This task is complicated by the fact that the extracellular signals may originate from one or more of the organisms that make up the coral holobiont which is composed of coral host (animal), symbiotic algae (both in the tissue and in the skeleton) and various microorganisms that reside in the coral mucus and tissues [20], [21]. Moreover, if extracellular enzymes are responsible for these signals, their interplay with intracellular processes may be rather complex. Nonetheless, given the importance (and danger) of ROS for cellular metabolism, these measurements may shed light on the chemical interactions between symbiotic algae and their coral hosts and between corals and other members of the reef community.

In the present study, a sensitive, rapid and selective chemiluminesence-based technique was adapted for quantifying superoxide production and detoxification (antioxidant activity) in the water surrounding the coral *Stylophora pistillata* and its symbiont zooxanthellae. Emphasis was placed on refining the method (including the conversion of signals to concentrations and rates) and establishing baseline values for non-stressed corals and algae, which may later be used for probing stress responses. In addition, the relative contribution of the various organisms composing the holobiont in superoxide production or destruction was assessed using cultured algae, bleached versus non-bleached corals, and metabolic inhibitors.

## Materials and Methods

The use of corals for this research was approved by the Israel Nature and Parks Authority.

### Trace metal clean techniques

All experimental manipulations were carried out in a clean room facility or under positive pressure HEPA filters. Plasticware was soaked in 10% HCl and then thoroughly rinsed in Milli-Q water. Solutions were prepared with 18.2 MΩ.cm Milli-Q water and reagent grade or higher purity salts. Gulf of Aqaba surface seawater was collected off a small fiberglass boat from open waters (bottom depth >300 m) and filtered in a clean room facility using a peristaltic pump (Cole-Parmer) and a capsule (Pall AcroPak) with a 0.2 µm supor membrane. A complete list of the reagents used and their preparation is outlined in the supplementary information (Reagents and Analysis S1).

### Superoxide measurements

#### Analysis and calibration

**Figure 1:**
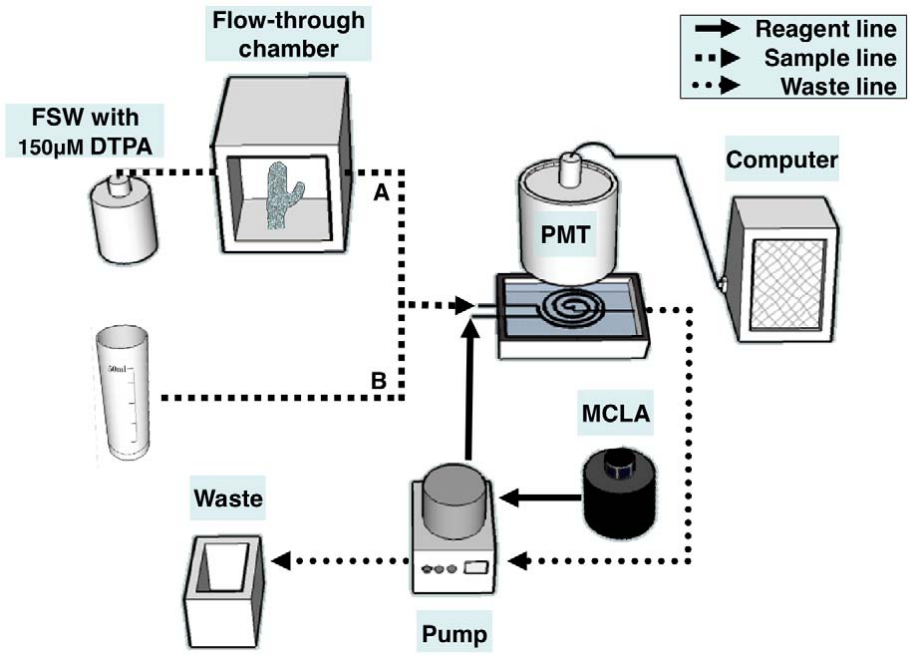
Setup for measuring extracellular superoxide production (A) and superoxide detoxification activity (B). (A). Coral O_2_^−^ production was measured using a temperature controlled stirred flow-through chamber and algal O_2_^−^ production was measured using an online syringe filter. Filtered seawater (FSW) was pumped through the chamber or the syringe filter and the O_2_^−^ signal was recorded downstream. (B). Coral O_2_^−^ detoxification activity was measured by adding O_2_^−^ spikes to seawater previously incubated with corals. Calibrations and controls were done in a similar manner with FSW. Both sample and reagent were drawn into a spiral flow cell at a rate of 5 mL min^−1^ and the emitted light was collected by a PMT and reported to a personal computer. To shorten the time O_2_^−^ spent in the line the sample was drawn directly into the flow cell while the reagent and waste lines were drawn with a peristaltic pump. To eliminate contamination by trace metals and prolong O_2_^−^ half life, the filtered seawater used for the measurements were amended (and pre-equilibrated) with 150 µM DTPA.

Superoxide concentrations were determined with the chemiluminescent reagent Methyl Cypridina Luciferin Analogue (MCLA) using a flow injection analysis system (FeLume II; Waterville Analytical). The focus here is on the application of this method for coral studies; the readers are referred to several recent papers on O_2_^−^ measurements in seawater and algae with the same technique [22], [23], [24], [25], [26]. The FeLume system was operated in continuous mode: sample and the reagent were continuously drawn into a Plexiglas spiral mixing cell at a rate of 5 mL min^−1^ and the emitted light was collected with a photomultiplier tube (PMT, H9319 Hamamatsu; Fig. 1). Due to the fast decay of O_2_^−^ it was essential to shorten the time the sample spent in the tubing. This was achieved by pumping the reagent and waste streams in such a way that the sample stream was drawn directly into the instrument without passing through the pump [22]. The system was calibrated with O_2_^−^ obtained from the UV photolysis of Benzophenon and 2-Propanol pH 12 cocktail using a mercury pan-lamp (Pen Ray; [27]; Fig. S1). Fresh cocktail was irradiated for each calibration and the resulting O_2_^−^ was measured in a UV-vis spectrophotometer (Cary Varian 50Bio) at 240nm (ε_240_ = 2345 M^−1^ cm^−1^, [28]). Each calibration curve was composed of 3–5 O_2_^−^ spikes at concentrations of 10–100 nM and took at most 10 min (Fig. S1, Reagents and Analysis S1). To account for superoxide decay between the spike addition and its detection in the FeLume (typically 20–30 sec, recorded for each spike), the signal was extrapolated to time zero (t_0_) by plotting the natural logarithm of the blank subtracted signal (Fig. S1).

#### Extracellular superoxide production

Extracellular O_2_^−^ production by corals was studied by placing coral nubbins in a 3 mL flow-through temperature controlled chamber with a built-in rotor circulating the water. Filtered seawater (FSW), amended with 150 µM of the metal chelator diethylenetriaminepentaacetic acid (DTPA), was pumped continuously through the chamber (Fig. 1) and the signal in the water leaving the chamber was measured and converted to O_2_^−^ concentrations using a daily established calibration curve (Fig. S1). Since O_2_^−^ also decays in the chamber, the calculated coral O_2_^−^ production rate (in units of mol O_2_^−^ min^−1^) includes a decay correction:
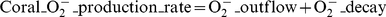
(1)

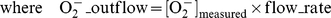


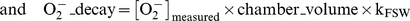
Where k_FSW_ refers to a pseudo first-order O_2_^−^ decay constant in filtered seawater (see next section). To allow comparison between different corals the O_2_^−^ production rates were further normalized to coral protein, surface area or volume. Identical chamber and flow rates were used for all reported measurements to minimize experimental variability and coral nubbin size was kept as similar as possible (typically 1–2 g with 0.8–1.2 mg protein/coral). The range of reported rates is based on 10 coral nubbins each measured 2–3 times in a single run.

Superoxide production by *Symbiodinium* cultures from clades A and C was measured in a setting parallel to that of the corals, using algae loaded onto 0.2 µm inline filter (that was thoroughly washed prior to analysis). O_2_^−^ production rates were calculated using Equation 2.
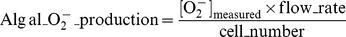
(2)This equation was validated by changing the number of cells loaded on to a filter. The range of reported rates is based on 15 filters loaded with algae that were measured twice in a single run. The effect of strong light (1500 µmol quanta m^−2^ s^−1^ white light, Schott ACEI), SOD and the NADPH Oxidase inhibitor Diphenyleneiodonium chloride (DPI, 6 µM) on O_2_^−^ production was examined for both corals and algae. Other details on these reagents and analyses are given in the supplementary information (Reagents and Analysis S1).

#### Extracellular superoxide detoxification

Superoxide detoxification was studied by following the decay kinetics of superoxide spikes in seawater that were incubated with the coral prior to analysis. In addition to reactions with the coral released antioxidant, the highly reactive O_2_^−^ may also decay by several other pathways, including acid-catalyzed disproportionation with its conjugate acid, to form H_2_O_2_ [29], reactions with trace metals [30] and reactions with dissolved organic matter (DOM, [31]). The overall (potential) decay of superoxide can then be described as:
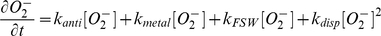
(3)Where the 1^st^ term is the coral extracellular antioxidant activity, the 2^nd^ term is the trace metals sinks, the 3^rd^ term is the non-trace metal sinks (possibly scavenging by DOM) and the 4^th^ term is the O_2_^−^ disproportionation (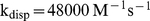 in seawater, [29]). To simplify the analysis the 2^nd^ term was eliminated by adding 150 µM DTPA to the seawater (at least 24 hrs prior to the analysis). DTPA forms complexes with trace metals that do not react with O_2_^−^ and hence it essentially masks the reactions between trace metals and O_2_^−^ [22], [31]. In addition, the 4^th^ term was significantly minimized by using low 10–100 nM O_2_^−^ spikes ([29], since this is a second-order process). Left with only the 1^st^ and 3^rd^ terms (where the latter is simply the decay of the water prior to the incubation), the O_2_^−^ decay in coral incubated seawater (CSW) is expected to follow a pseudo first-order, and the decay constant (k_CSW_) can be used for quantifying the antioxidant activity.

In each test, seawater incubated with a single coral nubbin for 20 min (CSW) was divided into 3–5 acid-cleaned test tubes. Each test tube was spiked with different O_2_^−^ concentrations and their decay kinetics were recorded. While each O_2_^−^ concentration decayed at a different rate, similar decay constants (k_CSW_) were obtained for all test tubes reinforcing the pseudo first-order behavior and improving analytical reproducibility. These pseudo first-order decay constants were calculated by plotting the natural logarithm of O_2_^−^ concentration over time (slope = k, Fig. S1B). Accordingly, the antioxidant activity released by corals to seawater is expressed herein as k_CSW_ (in units of s^−1^). In practice k_CSW_ also includes the background decay of O_2_^−^ in filtered seawater (k_FSW_) which was evaluated in each experiment, thus 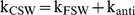. The extracellular antioxidant activity of *Symbiodinium* (clade C) was tested by loading 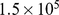, 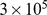 and 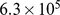 cells on single-use inline 0.2 µm filters (Minisart, Sartorius) and circulating them with FSW for 30 min, which was later spiked with O_2_^−^.

### Organisms and conditioning for experiments

#### Corals

*Stylophora Pistillata* fragments were collected from the coral reef near the Interuniversity Institute for Marine Sciences in Eilat (IUI), Red Sea, at 4 m depth. For each set of experiments 10–20 small fragments (0.45–0.55 cm long, 1–2 g) from the same colony were collected and grown in a 30% shaded open water system table for at least 1 month prior to measurements (midday light intensity of 400–600 µmol quanta m^−2^ s^−1^). Bleached corals were grown under dark conditions and were fed once a week. Corals were transferred gently to the lab prior to the experiments and maintained for a few hours in DTPA containing FSW under laboratory illumination. Experiments were run throughout the day as preliminary tests revealed no consistent effect of time of day on O_2_^−^ production. Protein concentrations were measured using a Bradford assay kit (Bio-Rad), with BSA as a standard, on coral tissues that had been removed from the skeleton by an air brush and homogenized.

#### Algae

Two *Symbiodinium* strains from clades C and A were chosen to represent the clades characterized from local *Stylophora pistillata* colonies [32]. The *Symbiodinium* strains *Symbiodinium goreaui* (CCMP 2466, clade C) and *Symbiodinium* sp. (CCMP 831, clade A) were grown on Guillard's f/2 medium (Sigma, diluted 1∶50 in sterile FSW). The cultures were kept in sterile, trace metal clean glass erlenmeyers fitted with a breathable cap at 24°C and under continuous light of 50 µmol quanta m^−2^ s^−1^. Cells were counted using a Beckman Z2 Coulter Counter, and specific growth rates were then determined from the linear regressions of the natural log of cell density versus time. Experiments were conducted during the exponential growth phase. In some experiments, aliquots of a single culture were acclimated for 24 hrs at 26°C or 32°C in the dark or in the light (300 µmol quanta m^−2^ s^−1^), loaded onto syringe filters and then tested under similar conditions. In other experiments, the short-term response of *Symbiodinium* loaded on syringe filters was tested with a strong light source (1500 µmol quanta m^−2^ s^−1^ white light, Schott ACEI). Note that the algae loaded onto syringe filters receive only a fraction of the source light and hence we refrain from specifying the exact light level they experienced.

### Statistics

General data manipulation, calculation of descriptive statistics, t-test and ANOVA were performed using SPSS (version 15.0). Cochran's test was used to test for homogeneity of variance prior to ANOVA; in cases of non-homogenous variance, either log10 or forth-root transformations were used (after confirming homogeneity of variance for the transformed data). Tukey's test was applied for post hoc analysis.

## Results and Discussion

### Methodology and concepts of studying superoxide dynamics in the coral's external *milieu*

**Figure 2:**
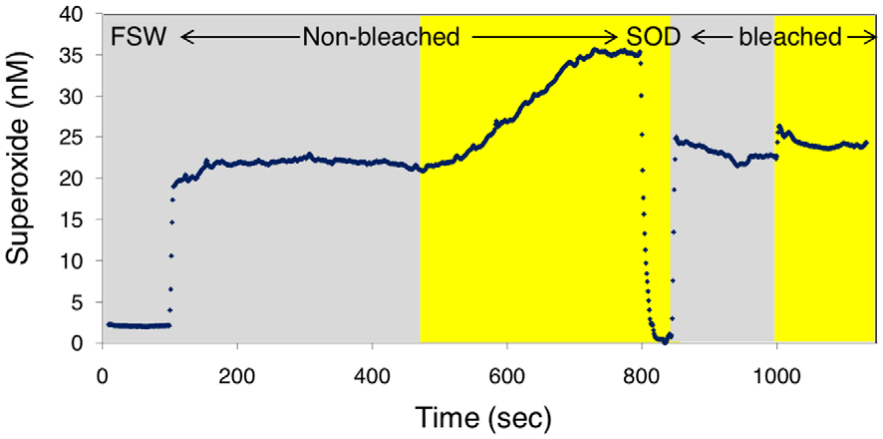
Typical FeLume run showing superoxide production by *Stylophora pistillata* nubbins. Here, two different *Stylophora* nubbins, bleached and non-bleached were tested. Each nubbin was measured first in the dark (grey background) and then illuminated by 1500 µmol quanta m^−2^ s^−1^ white light (yellow background). The run started by establishing the background signal of filtered seawater (FSW). Addition of superoxide dismutase (0.1 U/ml) at ∼800 sec resulted in a complete quench of the signal to that of FSW (and an even somewhat lower signal), confirming that the measured species is indeed superoxide.

**Figure 3:**
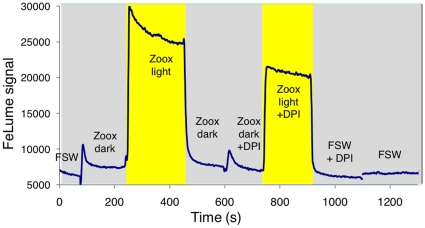
Typical FeLume run showing superoxide production by *Symbiodinium*. Here, *Symbiodinium sp.* from clade A (CCMP 831) loaded on a syringe filter was tested in the dark (grey background) and in the light (yellow background). Each run started and ended by recording the background signal of filtered seawater (FSW) flowing through an empty syringe filter. The effect of the NADPH Oxidase inhibitor DPI (6 µM added to FSW) on superoxide production by the algae and on the background FSW signal was also tested in the light and in the dark as labeled.

**Figure 4:**
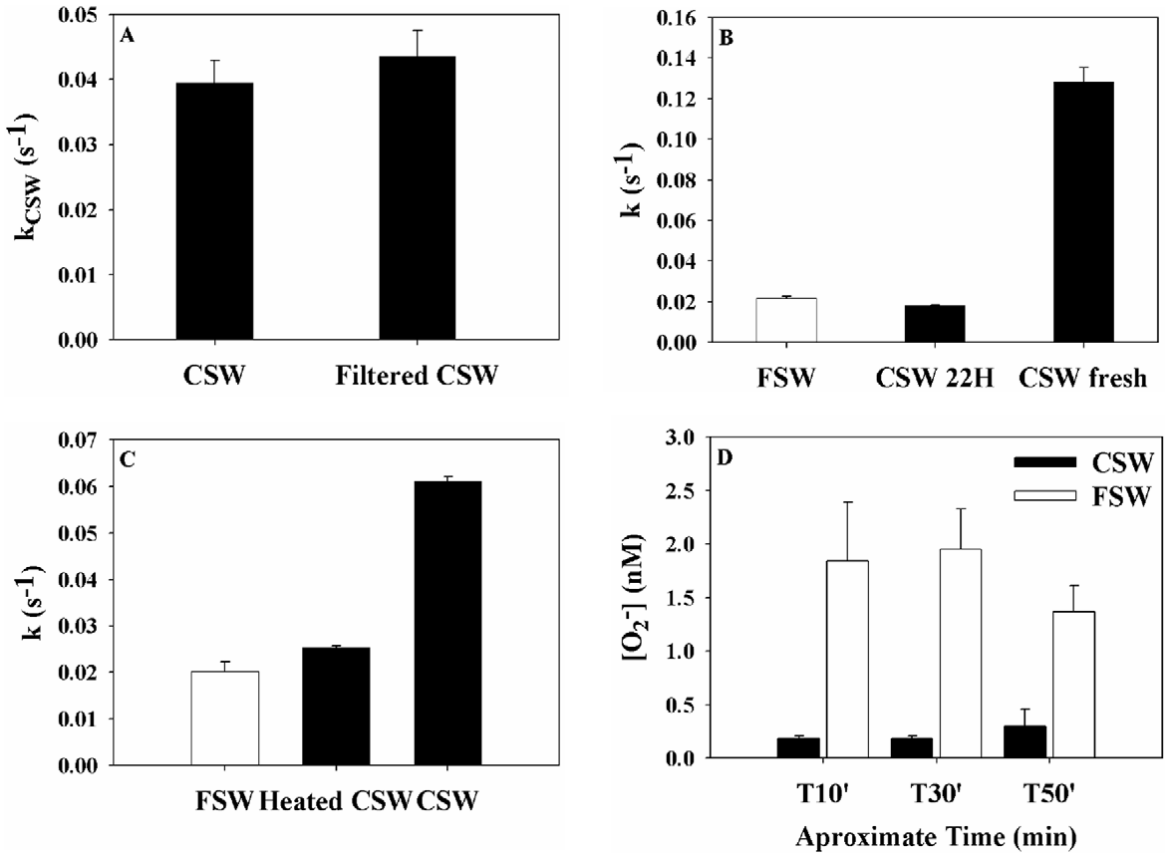
Assessment of coral O_2_^−^ detoxifying activity using photochemically generated superoxide spikes. (A). A composite of two FeLume runs in which 55 nM O_2_^−^ spikes were added to filtered seawater (FSW; open symbols) and to seawater previously incubated with a coral (CSW, closed symbols). Superoxide concentrations peaked when the spike reached the detector (∼at 15 sec) and then it decayed back to background values. The antioxidant released by the coral resulted in a faster decay of the O_2_^−^ spike as compared with FSW. (B). Same data as in A but plotted in a log-linear plot as the natural logarithm of [O_2_^−^] against time. The slopes of the lines stand for pseudo first-order O_2_^−^ decay constants (k_CSW_ or k_FSW_), which are used to quantify the antioxidant activity. (C). Effect of the coral dimensions (volume or weight) on the antioxidant activity it released (expressed as superoxide decay constant - k_CSW_). (D). Effect of incubation length on the antioxidant activity released by coral (expressed as superoxide decay constant - k_CSW_).

**Figure 5:**
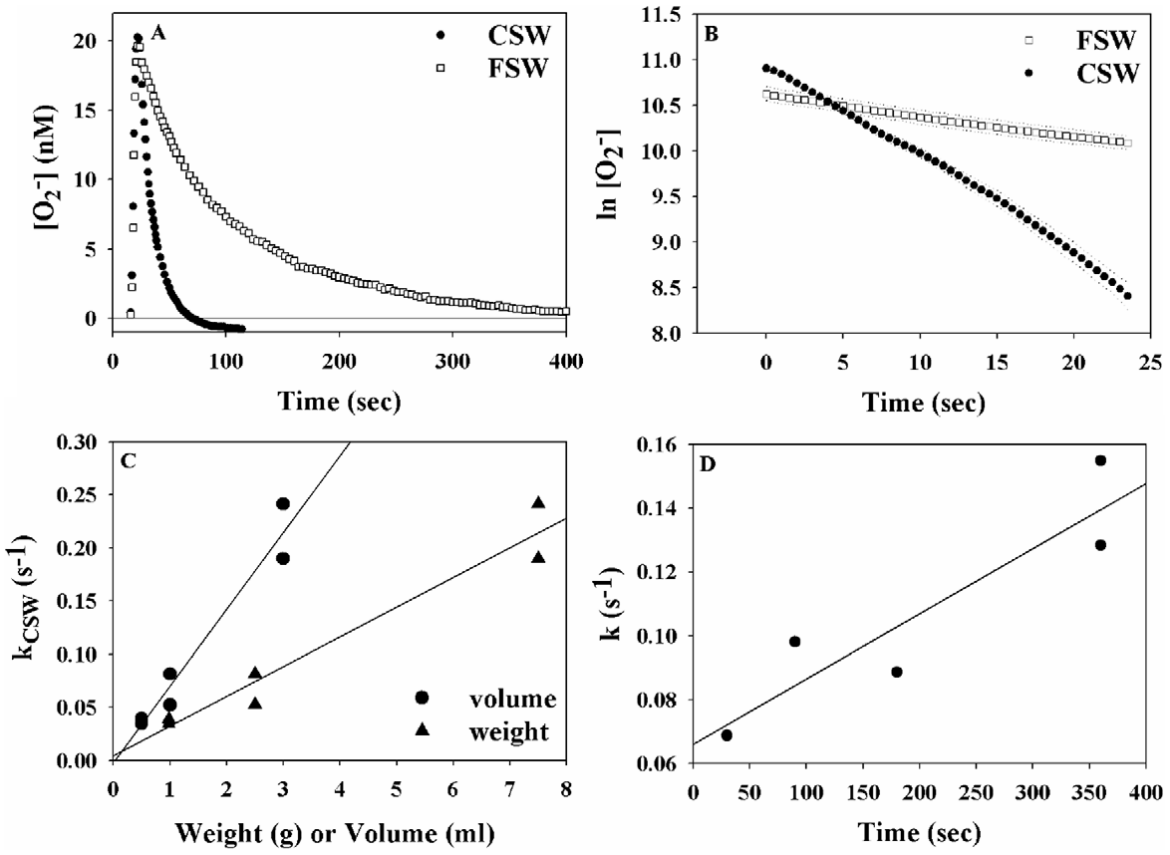
Partial biochemical characterization of the coral extracellular antioxidant activity, expressed as a pseudo first order-decay constant k_CSW_. (A). Filtration through 0.2 µm had no significant effect (*p>0.05*, student's t-test) on the coral antioxidant activity. (B). Storage of the CSW in the lab for 22 hrs resulted in a loss of the antioxidant activity to background levels (FSW). (C). Heating at 90°C for 20 min resulted in a loss of the antioxidant activity to background levels (FSW). For both B and C, the^−^ decay in CSW prior to storage or heating was significantly higher than the other treatments (*p<0.001*, one way ANOVA). (D). Steady-state O_2_^−^ concentrations measured over time in FSW and CSW in which O_2_^−^ was continuously produced by the Xanthine-Xanthine Oxidase system. Throughout the experiment the coral antioxidant activity maintained significantly lower O_2_^−^ concentrations as compared with FSW (*p<0.001* student's t-test), possibly showing that this activity is regenerated rather than saturated.

We applied a sensitive, rapid and selective chemiluminesence based technique for detecting sub-nanomolar superoxide concentrations in seawater to study the O_2_^−^ dynamics in *Stylophora pistillata* and its symbionts (Fig. 1). The chemiluminescent reagent, MCLA, is highly selective towards O_2_^−^ and is relatively insensitive to chemical interferences in seawater [22]. The system configuration allows rapid superoxide detection typically within 5–10 sec from the production source, be it a filter loaded with algae, a coral contained in a flow chamber, or a test tube spiked with O_2_^−^, which is essential for such a short-lived radical. Two types of processes were measured with this technique: extracellular O_2_^−^ production rates (Figs. 2 and 3, Tables 1 and 2) and extracellular O_2_^−^ detoxifying activity (antioxidant activity; Figures 4 and 5). The method's high sensitivity enables the probing of O_2_^−^ production by small coral fragments (0.5 cm long nubbins) and low microalgae densities (∼10^5^ cells), and the study of O_2_^−^ detoxifying activity at environmentally relevant nanomolar O_2_^−^ concentrations. At these low O_2_^−^ concentrations particular care is required to avoid contamination by trace metals that are likely to react with O_2_^−^. Coral studies are especially contamination prone since the pumps circulating seawater through water tables or aquariums where the corals are kept during acclimation are metal rich. In addition to applying trace metal clean techniques throughout all measurements were conducted in the presence of the strong chelator DTPA that masks trace metal interactions with O_2_^−^ [22], [24], [31]. As shown below large variations in O_2_^−^ production rates were found between corals fragments taken from the same colony, making the comparison between treatments difficult. This technique is most suitable for studying transient, short-term responses of a single coral fragment to environmental factors such as temperature and light or to metabolic inhibitors and metabolites. The other measurements of algal O_2_^−^ production and coral antioxidant activity are highly reproducible and, once mastered, are relatively easy. Both O_2_^−^ production and detoxification techniques are non-invasive and do not require that the coral be sacrificed. While some of the rates presented here are normalized to protein, normalization to coral surface area or volume yielded similar trends (data not shown), enabling the measurements to be non-destructive. Finally, the assessment of antioxidant activity involves only water collection and hence it can be applied *in situ*, in a natural coral reef.

**Table 1:** Extracellular superoxide production rates by *Stylophora pistillata*.

	Symbiont *Stylophora*					Aposymbiont (bleached) *Stylophora*				
Sample	I	II	III	IV	Average	I	II	III	IV	Average
Dark O_2_^−^ production rate* (nmol min^−1^ mg^−1^ protein)	0.95	0.27	0.19	0.41	**0.46 (0.34)**	0.34	0.20	0.74	1.41	**0.67 (0.54)**
Light O_2_^−^ production rate* (nmol min^−1^ mg^−1^ protein)	1.34	0.44	0.41	0.56	**0.69 (0.44)**	0.35	0.27	0.70	1.26	**0.64 (0.45)**
Light enhancement (%)**	41	63	115	35	**64^A^ (37)**	3	31	−5	−11	**5^B^ (19)**
DPI inhibition (%)***					**61 ; 76**					**76 ; 68**

*Superoxide production rates were calculated according to Equation 1 and normalized to protein.

**Calculated relative to the dark production rate ( = 100*(Light-Dark)/Dark).

***Calculated relative to the sample without DPI ( = 100*(with DPI- without DPI)/without DPI). Conducted in the light (right-hand side) and in the dark (left-hand side).

Superoxide production rates of symbiont (non-bleached) and aposymbiont (bleached) corals in the dark and in the light and with the NADPH Oxidase inhibitor DPI. Rates are presented for individual nubbins (samples I–IV) and as averages (1 SD in parentheses). An analysis of variance (ANOVA) between the symbiont and aposymbiont corals was performed for the dark and light treatments and for the light enhancement. Superscript letters indicate significantly different groupings; no letters indicate that the ANOVA test was not significant.

**Table 2:** Extracellular superoxide production rates by *Symbiodinium*.

	Clade C (CCMP 2466)		Clade A (CCMP 831)	
Temperature	26°C	32°C	26°C	32°C
Dark O_2_^−^ production rate* (fmol min^−1^cell^−1^)	0.282^A^ (0.062)	0.578^A^ (0.231)	0.048^X^ (0.036)	0.103^Y^ (0.035)
Light O_2_^−^ production rate* (fmol min^−1^cell^−1^)	0.952^B^ (0.179)	1.337^B^ (0.466)	0.062^X^ (0.041)	0.197^Z^ (0.018)
Light enhancement (%)**	241 (28)	138 (65)	36 (15)	101 (45)
DPI inhibition (%)***	32 ; 25			

*Superoxide production rates were calculated according to Equation 2. Data are means of 3–6 measurements conducted on different days.

**Calculated relative to the dark production rates ( = 100*(Light−Dark)/Dark).

***Effect of the NADPH Oxidase inhibitor DPI on O_2_^−^ production rate calculated relative to the sample without DPI ( = 100*(w/t DPI−without DPI)/without DPI). Conducted in the light (right-hand side) and in the dark (left-hand side), n = 2. The table does not include data from Figure 3.

Superoxide production rates of *Symbiodinium* cultures from clade C (CCMP 2466) and clade A (CCMP 831) which were subjected to the tested conditions of dark and light (300 µmol quanta m^−2^ s^−1^) at ambient temperature of 26°C and elevated temperature of 32°C for 24 hrs prior to and during the measurements. An analysis of variance (ANOVA) was performed separately for each *Symbiodinium* clade. Superscript letters indicate significantly different groupings.

### Coral and zooxanthellae extracellular O_2_^−^ production

All tested *Stylophora* nubbins continuously produced or released superoxide to the surrounding water at a rate of 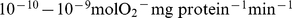 in the dark (Table 1, Fig. 2). Superoxide dismutase (SOD) addition resulted in an immediate drop of the signal to background levels, and even to slightly lower values (Fig. 2, Fig. S2), indicating that the chemical species we measure is indeed O_2_^−^. Rates obtained from replicate coral fragments taken from the same colony were highly variable, possibly reflecting physiological variations among corals, changes in the coral state, or imperfect experimental setup. We thus report the rates of each of the fragments individually and focus on their response to light or diphenyleneiodonium chloride (DPI) (Table 1). DPI is a membrane permeable inhibitor of transmembrane oxidoreductases such as the NADPH Oxidase (NOX) complex which reduces oxygen to superoxide.

Superoxide production from symbiotic (non-bleached) and aposymbiotic (bleached) corals was statistically indistinguishable in the dark (Table 1, 2 ways ANOVA *p>0.05*). Light was found to enhance O_2_^−^ production in non-bleached corals, resulting in a doubling of the rates (Table 1, Fig. 2; 2 ways ANOVA *p<0.01*). In contrast, light had no consistent effect on bleached corals (Table 1, Fig. 2; 2 ways ANOVA *p>0.05*). DPI had a strong inhibitory effect on O_2_^−^ production in non-bleached and bleached corals in the light and in the dark (Table 1).

Both *Symbiodinium* clades A and C were found to produce O_2_^−^ in the dark. Slower O_2_^−^ production rates of 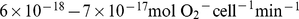 were recorded for clade A and higher rates of 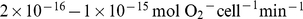 were found for clade C (Table 2, Fig. 3; 2ways ANOVA *p<0.001*). Substantially higher O_2_^−^ production was recorded when the algae were illuminated, already at low light levels (data not shown) and more so at higher light levels (Table 2, Fig. 3). Illumination caused an immediate enhancement of the O_2_^−^ flux by up to ten-fold (Fig. 3), a response that was faster and more pronounced than that of non-bleached corals (Fig. 2). Algae incubated for 24 hrs in the light released more O_2_^−^ than dark incubated algae (Table 2; 2ways ANOVA p<0.001), although the light effect was weaker than that of short-term illumination (Table 2, Fig. 3). An elevated water temperature of 32°C (4°C above the summer maximum), applied 24 hrs prior to and during the measurements had a significant effect on O_2_^−^ production by clade A only (Table 2; 2ways ANOVA p<0.01). The NOX inhibitor DPI, resulted in a moderate inhibition of O_2_^−^ production by *Symbiodinium* (Table 2, Fig. 3). Analytically, these measurements were much more reproducible than those with corals, possibly due to sample homogeneity and the use of a filter with a small volume as compared with the coral flow-through chamber (Table 2, SD on rates up to 20% in dark).

To the best of our knowledge this is the first paper to report *in vivo* production of key ROS species, the superoxide radical in intact *Stylophora* corals and its zooxanthellae (in culture). Two previous studies examined the production of O_2_^−^ by the photosymbiont containing sea anemone *Aiptasia pulchella* and *Anthopleura elegantissima* [33], [34]. Dykens et al. (1992) applied an electron paramagnetic resonance technique on tissue homogenates and found light dependent O_2_^−^ production that was linked to photosynthesis, as well as host mitochondrial O_2_^−^ production [34]. Nii and Muscatine (1997) examined the production of superoxide ions within sea anemone tissues using cytochrome c and nitro blue tetrazolium (NBT) reduction [33]. Both studies assigned the bulk of O_2_^−^ production to the sea anemone host as no O_2_^−^ release from freshly isolated *Symbiodinium* was detected [33]. Considering our data on O_2_^−^ release from *Symbiodinium* and the prevalence of this process among phytoplankton [35], it is likely that the sea anemone symbionts also produce O_2_^−^ which was not detected by the NBT technique. While we have no coral data for comparison, our measured algal O_2_^−^ production rates agree well with rates reported for the centric marine diatom *Thalassiosira Weissflogii* of 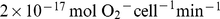 [23], [26]. These studies reported a strong inhibitory effect of DPI on O_2_^−^ production by diatoms, while we observed only a moderate inhibitory effect on *Symbiodinium*. This discrepancy probably reflects the much higher DPI concentrations used in those studies - concentrations which exceed the recommended dose of this inhibitor [36].

The light-enhanced superoxide production observed in non-bleached corals and in *Symbiodinium*, may hint at links between the two. Quantitatively, there are enough algae in the coral tissue to account for the observed O_2_^−^ production rates of the studied corals. The number of *Symbiodinium* cells in each nubbin was estimated as 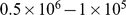 (surface area is 1–2 cm^2^ with 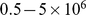 cells per cm^2^ [37], [38]). Multiplying these cell numbers by an algal O_2_^−^ production rate of 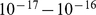 resulted in a production rate of 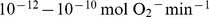. This rate is comparable to that obtained from *Stylophora* nubbins (that contain ∼1 mg protein/nubbin). Nonetheless, it highly questionable whether the short-lived charged O_2_^−^ molecules produced by *Symbiodinium* within the host tissue can cross several membranes and end up in the coral's external *milieu*. Moreover, bleached corals did produce O_2_^−^ at comparable rates to those of non-bleached corals in the dark, suggesting that at least some of the O_2_^−^ production is mediated by the host. While more research is needed to explore the biochemical pathways involved in the O_2_^−^ production, the strong inhibitory effect of DPI in corals (70–80% inhibition in bleached and non-bleached, Table 1) possibly indicates the importance of the NADPH Oxidase (NOX) pathway of the host. *Stylophora* most likely contains NOX akin to other cnidarians such as the sea anemone *Nematostella vectensis* [39], which are localized in the plasma membrane and the mitochondria.

In the light, S*ymbiodinium* seems to play a role in the holobiont O_2_^−^ production, either directly or indirectly, by generating reductive power and/or oxygen which boosts the host NOX pathway. Elevated superoxide production from NADPH Oxidases was reported for mammalian cells subjected to high oxygen levels (termed hyperoxia, [40]). As for *Symbiodinium*, the light-enhanced O_2_^−^ production is probably linked to their photosynthetic activity, while the NOX pathway is secondary as seen by the mild effect of DPI (∼20–30% inhibition of O_2_^−^ production rates, Table 2). In a recent paper by Suggett et al. (2008) H_2_O_2_ production (and release to the medium) by two *Symbiodinium* types was measured at varying temperature and light intensities [41]. H_2_O_2_ production was detected under ambient and stress conditions at rates ranging from 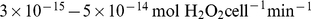 and was attributed to mitochondrial respiration in the dark and to Mehler reactions and mitochondrial alternative oxidases (AOX) in the light [41]. At high temperature the thermally sensitive type was found to have elevated light-driven H_2_O_2_ production, similar to our findings with clade A (Table 2). It is tempting to link the H_2_O_2_ fluxes to the somewhat lower superoxide production rates measured here for *Symbiodinium*. Nonetheless, as O_2_^−^ and H_2_O_2_ differ in their chemical reactivity and charge their extracellular fluxes may originate at least in part from distinct pathways.

### Coral extracellular superoxide detoxifying activity

*Stylophora Pistillata* incubated in filtered seawater at ambient temperature and low light was found to continuously release an antioxidant capable of detoxifying O_2_ into its surroundings. We examined this antioxidant activity by following the decay kinetics of photochemically generated O_2_^−^ spikes added to seawater, in which coral were previously incubated for 20 min (CSW, Figs. 4 and 5). Superoxide decay exhibited pseudo first-order kinetics (Figs. 4A and 4B, Table S1) and the O_2_^−^ decay constants (obtained by plotting the natural log of superoxide against time) were concentration independent as expected for a first-order reaction (Fig. S1B). In all cases tested (n = 69), higher antioxidant activity, expressed by a higher O_2_^−^ decay constant, was found in coral incubated water (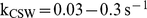) as compared with the non-incubated seawater (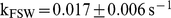, Fig. S3). Larger corals and longer incubation times resulted in a linear increase of k_CSW_ (Figs. 4C and 4D) although no correlation was found with coral protein content (data not shown). Similar antioxidant activity was seen for bleached and non-bleached corals in the dark and in laboratory light. Both clades of *Symbiodinium* showed no measurable effect on O_2_^−^ decay rates (using ∼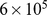 cells incubated for 30 minutes, Table S1), possibly suggesting that the symbiotic algae play a negligible role in the coral extracellular antioxidant activity.

While the antioxidant agent was not isolated, several experimental lines of evidence support its identification as an SOD-like enzyme/s (rather than non-specific long-lasting organic molecules). It is filterable (<0.2 µm, Fig. 5A), unstable with time (activity was lost during 22 hrs of storage at room temperature, Fig. 5B) and heat sensitive (activity was lost during 20 min at 90°C; Fig. 5C). Moreover, its decay kinetics resemble that of SOD [3], and its effect on the FeLume signal is similar to that of commercial SOD (CSW and SOD yield lower background signals as compared to FSW; Figs. 2 and S2). No titration or consumption of the coral released antioxidant activity was observed in experiments where O_2_^−^ was continuously generated by the Xanthine-Xanthine Oxidase system (Fig. 5D), an observation in support of renewable enzymatic pool, such as SOD.

Other proteins and small molecules could potentially account for the observed superoxide detoxification activity (Figs. 4 and 5). For instance, the ubiquitous green fluorescent proteins (GFP) were shown to quench superoxide [42]. As GFPs are unlikely to be released in large quantities to the surrounding waters their contribution to the observed extracellular activity is probably minor. Mycosporine-like amino acids (MAAs) that serve to protect corals from UV irradiation were also assigned antioxidant properties [43]. The small MMAs can be released to the water, but they were shown to be chemically stable (and photostable) over 24 hrs [44], unlike the antioxidant activity studied here (Fig. 5). The algal osmolyte dimethylsulphoniopropionate (DMSP) and its enzymatic cleavage product dimethylsulphide (DMS) were found to readily scavenge hydroxyl radicals and other reactive oxygen species, and thus may serve as an antioxidant system [45]. Significant concentrations of DMSP and DMS were recorded in coral reef organisms harboring symbiotic dinoflagellates, such as scleractinian corals and in their surroundings water (e.g. [46]). Nonetheless, this class of compounds does not play a role in the antioxidant activity observed here as *Symbiodinium*, the source of DMSP/DMS to the coral holobiont, had no measurable extracellular superoxide detoxification activity (Table S1).

Assuming that superoxide dismutase (SOD) is the primary source of the superoxide detoxifying activity released by the corals and expressed as k_CSW_, it can be converted to SOD activity by spiking O_2_^−^ to FSW amended with increasing concentrations of commercial SOD (Fig. S4, Reagents and Anaylsis S1). This exercise revealed that the antioxidant activity released from individual coral nubbins during 20 min of incubation is equivalent to SOD activities of 10–20 mU/ml. These low activities represent only the fraction released to the water, while the total extracellular coral antioxidant activity is potentially much greater.

The SOD-like activity may be generated by the coral host itself or its mucus-associated microorganisms (no activity was found for *Symbiodinium*, Table S1). Recently, extracellular SOD-like activity was measured in several coral-associated Vibrio species and was suggested to aid in detoxifying O_2_^−^ [47], [48]. Extracellular SOD was also reported to occur in cnidarians, and it was suggested to be released into the coelentera cavity and/or be trapped within the mucus produced by cnidarians [49], [50]. In the oxygen-rich strongly irradiated reef environment, SOD-like enzymes may serve to protect the coral holobiont (mostly the host and mucus associated microorganisms) from external O_2_^−^ fluxes. Alternatively, the antioxidant activities may represent intracellular processes of the host, such as oxidative stress (although our measurements were done under non-stressed conditions). More research is required to clarify these issues.

### Conclusions and implications

Our study places a strong emphasis on developing quantitative methods for obtaining O_2_^−^ production and detoxification rates in the external *milieu* of the coral *Stylophora pistillata* and cultured *Symbiodinium* under non-stress conditions. We found, however, increased O_2_^−^ production by cultured *Symbiodinium* in response to the known oxidative stress inducers, light and elevated temperature (to a smaller degree). We thus believe that our approach may be useful for oxidative stress studies in corals, mostly in combination with other molecular, biochemical and physiological measures of coral responses to ambient or stress conditions. Given the complex nature of the coral consortium (holobiont), many questions are still open as to the applications of our findings. It is unclear whether the *Stylophora* measured O_2_^−^ fluxes represent intra- or extra-cellular processes (or a combination) and whether it can serve as a proxy for stress response. The same applies to the extracellular O_2_^−^ detoxifying activity, which may reflect the host defense against elevated O_2_^−^ levels internally or externally (or a combination of both). In any event, the findings of extracellular O_2_^−^ production by *Symbiodinium* potentially place the algae as the major source of reactive oxygen radicals under light conditions within this symbiosis. If intact *Symbiodinium* (within the host) also produce O_2_^−^ and if it gets to the coral tissue, then our data imply that algal-bearing corals are more susceptible to an internal build-up of superoxide and possible apoptosis under severe environmental stress as suggested by numerous groups [9], [51], [52], [53].

## Supporting Information

Reagents and Analysis S1 (0.02 MB DOCX)

Table S1 (0.01 MB DOCX)

Figure S1 Calibration curve for superoxide in the Felume with MCLA. (A) Superoxide spikes at decreasing concentrations were added to FSW (with 150 µM DTPA) and the decay over time was recorded. (B) The decay curves were plotted in a log linear graph, allowing for back extrapolation to the original signal when the spike was added - t_0_ (it took ∼30 sec from spike addition to its detection). As expected from pseudo first-order decay kinetics, the O_2_^−^ decay curves are linear and their slopes equal to the O_2_^−^ decay constant (k_FSW_). (C). The end product calibration curve of the FeLume MCLA signal at time zero (t_o_) versus the concentrations of O_2_^−^ added. All calibration lines were linear (R^2^ >0.9) for superoxide range used, typically 20–80 nM. However, these lines do not go through the origin, probably because the MCLA response is concentration-dependent over larger ranges. This notion is supported by a second calibration curve conducted with sub nanomolar O_2_^−^, which crosses through the origin and has a higher slope.
(4.20 MB TIF)

Figure S2 Effect of superoxide dismutase (SOD) on the background signal of MCLA in the presence of superoxide free filtered seawater (FSW, donated by the error). SOD additions even at minimal levels caused a significant quenching of the chemiluminescence signal as was previously reported by Koga and Nakano, 1992 [4], and suggested to result by direct interaction of the enzyme with MCLA or its intermediate derivatives. Filtered seawater incubated with corals (CSW) also had lower background signal compared with FSW, further supporting our hypothesis that the detoxifying agent released from the corals resembles SOD. The detoxifying activity in CSW was converted to SOD units according to the calibration presented in Figure S4. The data is an average of 150 seconds of steady state signal where the standard deviations are too small to be seen.
(1.50 MB TIF)

Figure S3 Compilation of all superoxide decay constants (k_FSW_) in DTPA containing filtered seawater (FSW) obtained with O_2_^−^ spikes at different concentrations. Superoxide decay constants were independent of the spike concentrations (R^2^ = 0.199), indicating that the reaction is pseudo first-order and reaffirming our data analysis approach (Fig. S1). The dotted line represents the average superoxide decay constant in FSW, which serves as a background for the coral induced elevated O_2_^−^ decays. The grey area represents 1 standard deviation (1SD) on the average.
(6.01 MB TIF)

Figure S4 Calibration between the activity of commercial SOD and superoxide decay rates (expressed as pseudo first order decay constant k). Having established that the coral antioxidant activity resembles that of SOD (Figure 5), this curve enables the conversion of the experimentally measured superoxide decay rates to SOD activity, as done in Figure S2.
(1.70 MB TIF)

## References

[1] Byczkowski JZ, Gessner T (1988). Biological role of superoxide ion radical.. International Journal of Biochemistry.

[2] Fattman CL, Schaefer LM, Oury TD (2003). Extracellular superoxide dismutase in biology and medicine.. Free Radical Biology and Medicine.

[3] Fridovich I (1983). Superoxide radical - An endogenous toxicant.. Annual Review of Pharmacology and Toxicology.

[4] Lesser MP (2006). Oxidative stress in marine environments: Biochemistry and physiological ecology.. Annual Review of Physiology.

[5] Smith DJ, Suggett DJ, Baker NR (2005). Is photoinhibition of zooxanthellae photosynthesis the primary cause of thermal bleaching in corals?. Global Change Biology.

[6] Weis VM (2008). Cellular mechanisms of Cnidarian bleaching: stress causes the collapse of symbiosis.. Journal of Experimental Biology.

[7] Murata N, Takahashi S, Nishiyama Y, Allakhverdiev SI (2007). Photoinhibition of photosystem II under environmental stress.. Biochimica et Biophysica Acta-Bioenergetics.

[8] Glynn PW (1996). Coral reef bleaching: Facts, hypotheses and implications.. Global Change Biology.

[9] Richier S, Sabourault C, Courtiade J, Zucchini N, Allemand D (2006). Oxidative stress and apoptotic events during thermal stress in the symbiotic sea anemone, *Anemonia viridis*.. FEBS Journal.

[10] Richier S, Rodriguez-Lanetty M, Schnitzler CE, Weis VM (2008). Response of the symbiotic cnidarian *Anthopleura elegantissima* transcriptome to temperature and UV increase.. Comparative Biochemistry and Physiology D-Genomics & Proteomics.

[11] Brown BE, Downs CA, Dunne RP, Gibb SW (2002). Exploring the basis of thermotolerance in the reef coral *Goniastrea aspera*.. Marine Ecology-Progress Series.

[12] Downs CA, Mueller E, Phillips S, Fauth JE, Woodley CM (2000). A molecular biomarker system for assessing the health of coral (*Montastraea faveolata*) during heat stress.. Marine Biotechnology.

[13] Flores-Ramirez LA, Linan-Cabello MA (2007). Relationships among thermal stress, bleaching and oxidative damage in the hermatypic coral, *Pocillopora capitata*.. Comparative Biochemistry and Physiology C-Toxicology & Pharmacology.

[14] Fitt WK, Gates RD, Hoegh-Guldberg O, Bythell JC, Jatkar A (2009). Response of two species of Indo-Pacific corals, P*orites cylindrica* and *Stylophora pistillata*, to short-term thermal stress: The host does matter in determining the tolerance of corals to bleaching.. Journal of Experimental Marine Biology and Ecology.

[15] Lesser MP (1996). Elevated temperatures and ultraviolet radiation cause oxidative stress and inhibit photosynthesis in symbiotic dinoflagellates.. Limnology and Oceanography.

[16] Sandeman IM (2006). Fragmentation of the gastrodermis and detachment of zooxanthellae in symbiotic cnidarians: a role for hydrogen peroxide and Ca^2+^ in coral bleaching and algal density control.. Revista de Biologia Tropical (San Jose).

[17] Bartosz G (2006). Use of spectroscopic probes for detection of reactive oxygen species.. Clinica Chimica Acta.

[18] Bonini MG, Rota C, Tomasi A, Mason RP (2006). The oxidation of 2 ′,7 ′-dichlorofluorescin to reactive oxygen species: A self-fulfilling prophesy?. Free Radical Biology and Medicine.

[19] Chignell CF, Sik RH (2003). A photochemical study of cells loaded with 2 ′,7 ′-dichlorofluorescin: Implications for the detection of reactive oxygen species generated during UVA irradiation.. Free Radical Biology and Medicine.

[20] Wegley L, Edwards R, Rodriguez-Brito B, Liu H, Rohwer F (2007). Metagenomic analysis of the microbial community associated with the coral *Porites astreoides*.. Environmental Microbiology.

[21] Rosenberg E, Koren O, Reshef L, Efrony R, Zilber-Rosenberg I (2007). The role of microorganisms in coral health, disease and evolution.. Nature Reviews Microbiology.

[22] Rose AL, Moffett JW, Waite TD (2008a). Determination of superoxide in seawater using 2-methyl-6-(4-methoxyphenyl)-3,7-dihydroimidazo[1,2-a]pyrazin-3(7H)-one chemiluminescence.. Analytical Chemistry.

[23] Rose AL, Webb EA, Waite TD, Moffett JW (2008b). Measurement and implications of nonphotochemically generated superoxide in the equatorial Pacific Ocean.. Environmental Science and Technology.

[24] Shaked Y, Harris R, Klein-Kedem N (2010). Hydrogen peroxide photocycling in the Gulf of Aqaba, Red Sea.. Environmental Science and Technology.

[25] Milne A, Davey MS, Worsfold PJ, Achterberg EP, Taylor AR (2009). Real-time detection of reactive oxygen species generation by marine phytoplankton using flow injection-chemiluminescence.. Limnology and Oceanography-Methods.

[26] Kustka AB, Shaked Y, Milligan AJ, King DW, Morel FMM (2005). Extracellular production of superoxide by marine diatoms: Contrasting effects on iron redox chemistry and bioavailability.. Limnology and Oceanography.

[27] McDowell MS, Bakac A, Espenson JH (1983). A convenient route to superoxide ion in aqueous-solution.. Inorganic Chemistry.

[28] Bielski BHJ (1978). Re-evaluation of spectral and kinetic properties of HO_2_ and O_2_
^−^ free radicals.. Photochemistry and Photobiology.

[29] Bielski BHJ, Cabelli DE, Arudi RL, Ross AB (1985). Reactivity of HO_2_/O_2_
^−^ radicals in aqueous solution.. Journal of Physical and Chemical Reference Data.

[30] Rush JD, Bielski BHJ (1985). Pulse radiolytic studies of the reactions of HO_2_/O_2_
^−^ with Fe(II)/Fe(III) ions - The reactivity of HO_2_/O_2_
^−^ with ferric ions and its implication on the occurrence of the Haber-Weiss reaction.. Journal of Physical Chemistry.

[31] Goldstone JV, Voelker BM (2000). Chemistry of superoxide radical in seawater: CDOM associated sink of superoxide in coastal waters.. Environmental Science and Technology.

[32] Winters G, Beer S, Ben Zvi B, Brickner I, Loya Y (2009). Spatial and temporal photoacclimation of *Stylophora pistillata*: zooxanthella size, pigmentation, location and clade.. Marine Ecology-Progress Series.

[33] Nii CM, Muscatine L (1997). Oxidative stress in the symbiotic sea anemone *Aiptasia pulchella* (Carlgren, 1943): Contribution of the animal to superoxide ion production at elevated temperature.. Biological Bulletin.

[34] Dykens JA, Shick JM, Benoit C, Buettner GR, Winston GW (1992). Oxygen radical production in the sea-anemone *Anthopleura Elegantissima* and its endosymbiotic algae.. Journal of Experimental Biology.

[35] Marshall JA, de Salas M, Oda T, Hallegraeff G (2005). Superoxide production by marine microalgae.. Marine Biology.

[36] Heyno E, Klose C, Krieger-Liszkay A (2008). Origin of cadmium-induced reactive oxygen species production: mitochondrial electron transfer versus plasma membrane NADPH oxidase.. New Phytologist.

[37] Hoegh-Guldberg O, Smith GJ (1989). The effect of sudden changes in temperature, light and salinity on the population density and export of zooxanthellae from the reef corals *Stylophora Pistillata Esper* and S*eriatopora-Hystrix Dana*.. Journal of Experimental Marine Biology and Ecology.

[38] Porter JW, Muscatine L, Dubinsky Z, Falkowski PG (1984). Primary production and photoadaptation in light-adapted and shade-adapted colonies of the symbiotic coral, *Stylophora Pistillata*.. Proceedings of the Royal Society of London Series B-Biological Sciences.

[39] Sumimoto H (2008). Structure, regulation and evolution of Nox-family NADPH oxidases that produce reactive oxygen species.. FEBS Journal.

[40] Jamieson D, Chance B, Cadenas E, Boveris A (1986). The relation of free-radical production to hyperoxia.. Annual review of physiology.

[41] Suggett DJ, Warner ME, Smith DJ, Davey P, Hennige S (2008). Photosynthesis and production of hydrogen peroxide by *Symbiodinium* (Pyrrhophyta) phylotypes with different thermal tolerances.. Journal of Phycology.

[42] Bou-Abdallah F, Chasteen ND, Lesser MP (2006). Quenching of superoxide radicals by green fluorescent protein.. Biochimica et Biophysica Acta-General Subjects.

[43] Dunlap WC, Yamamoto Y (1995). Small-molecule antioxidants in marine organisms - Antioxidant activity of Mycosporine-Glycine.. Comparative Biochemistry and Physiology B-Biochemistry & Molecular Biology.

[44] Whitehead K, Hedges JI (2005). Photodegradation and photo sensitization of mycosporine-like amino acids.. Journal of Photochemistry and Photobiology B-Biology.

[45] Sunda W, Kieber DJ, Kiene RP, Huntsman S (2002). An antioxidant function for DMSP and DMS in marine algae.. Nature.

[46] Raina JB, Dinsdale EA, Willis BL, Bourne DG (2010). Do the organic sulfur compounds DMSP and DMS drive coral microbial associations?. Trends in Microbiology.

[47] Banin E, Vassilakos D, Orr E, Martinez RJ, Rosenberg E (2003). Superoxide dismutase is a virulence factor produced by the coral bleaching pathogen *Vibrio shiloi*.. Current Microbiology.

[48] Munn CB, Marchant HK, Moody AJ (2008). Defenses against oxidative stress in vibrios associated with corals.. FEMS Microbiology Letters.

[49] Dash B, Metz R, Huebner HJ, Porter W, Phillips TD (2007). Molecular characterization of two superoxide dismutases from *Hydra vulgaris*.. Gene.

[50] Plantivaux A, Furla P, Zoccola D, Garello G, Forcioli D (2004). Molecular characterization of two CuZn-superoxide dismutases in a sea anemone.. Free Radical Biology and Medicine.

[51] Dunn SR, Thomason JC, Le Tissier MDA, Bythell JC (2004). Heat stress induces different forms of cell death in sea anemones and their endosymbiotic algae depending on temperature and duration.. Cell Death and Differentiation.

[52] Lesser MP, Farrell JH (2004). Exposure to solar radiation increases damage to both host tissues and algal symbionts of corals during thermal stress.. Coral Reefs.

[53] Tchernov D, Gorbunov MY, de Vargas C, Yadav SN, Milligan AJ (2004). Membrane lipids of symbiotic algae are diagnostic of sensitivity to thermal bleaching in corals.. Proceedings of the National Academy of Sciences of the United States of America.

