# Supplementary material for: Extracellular Production and Degradation of Superoxide in the Coral Stylophora pistillata and Cultured Symbiodinium
Source: PLoS One. 2010 Sep 14;5(9):e12508. doi: 10.1371/journal.pone.0012508 (PMC2939047; doi:10.1371/journal.pone.0012508)
Supplement: Reagents and Analysis S1 — (0.02 MB DOCX) [file pone.0012508.s001.docx]

**Reagents and Analysis - Supporting Information**

***Reagents –***

- The MCLA working solution consisted of 50 mM sodium acetate (Sigma) buffer, 150 μM diethylenetriaminepentaacetate (DTPA; Sigma), and 1 μM 2-methyl-6-(*p*-methoxyphenyl)-3,7-dihydroimidazo[l,2-a]pyrazin-3-one hydrochloride (MCLA, fluka). The solution pH was set to 6 with double distilled HCl and it was stored at 4^0^C in a dark polycarbonate bottle and used for up to a couple of weeks. Upon receipt, MCLA (10 mg) was dissolved in 14 mL DDW and divided into small aliquots and kept frozen at -80°C.
- The photolysis cocktail was made fresh before use by mixing 6.2 ml pH 12 NaOH solution (20 mM, Merck), 3.8 ml of 2-propanol (99.8%, Merck) and 63 μl of 1.1 mM benzophenon solution (Merck). Benzophenon stock solution was made by dissolving it first in 2-propanol and then adding mili-Q water.
- The mammalian NADPH Oxidase (NOX) inhibitor Diphenyleneiodonium chloride (DPI Calbiochem #300260, recommended dosage < 10μM [1]), was used for characterizing superoxide sources. Fresh DPI solution was added to the solution flashing the coral at a final concentration of 6.35 μM.
- Bovine SOD (Calbiochem #574593) diluted in DDW to a stock concentration of 1500 U ml^-1^, was used as a positive control for antioxidant activity and for calibrating the CSW decay to SOD units.
- The xanthine oxidase (Sigma 115k37902) enzyme which mediates the formation of superoxide through the oxidation of xanthine (Sigma) was used at concentrations of 0.04 U l^-1^ and 16 μM, respectively).

***Method validation and artifact elimination***

- To verify that the coral and algal generated signal was indeed superoxide we applied SOD to the feeding solution, which resulted in an immediate drop of the signal followed by a slow recovery as the enzyme was washed away (Fig. 2). Another demonstration that the measured species is a fast decaying radical and not an algal induced artifact, was achieved by increasing the tube length and hence the time between the algal loaded filter and the FeLume detector, which resulted in gradually lower signals.
- To probe for the importance of NOX in the measured superoxide flux we have added the NOX inhibitor DPI, which decreases the reduction of its flavin redox center [2], to the filtered seawater that were circulated through the corals and algae. We have first verified that DPI does not react with O_2_^-^ and/or MCLA nor generates chemical artefacts by examining its effect on the background signal and on photochemically generated O_2_^•-^ spike in the absence of a biological sample (data not shown). In addition, we insured that the DPI effect on the organisms was reversible by running FSW through the treated sample for 30 more minutes until the original signal was obtained.

***Superoxide calibration curves (in detail)***

Superoxide spikes were obtained from UV photolysis of 63 μM Benzophenon in 5 M 2-Propanol prepared in dilute NaOH at pH 12 (referred hereafter as cocktail). Fresh cocktail was made daily in a test-tube and was shaken vigorously prior to irradiation (to oxygenate it). Four mL of this cocktail was transferred to a 1 cm quartz cuevate and irradiated for 10-30 sec with a mercury pan-lamp (Pen Ray). The resulting 1-10 μM O_2_^-^ was monitored in a UV-vis spectrophotometer (Cary Varian 50Bio) at 240nm (ε_240_=2345M^-1^cm^-1^, [3]). The cocktail has a significant absorbance at 240nm, and it was assessed carefully before illumination by repeated scans. Each spectrophotometer reading included a scan from 200 to 400nm and the absorption at 400nm was subtracted from the desired adsorption at 240nm in all readings (to correct for "floating" spectra). O_2_^-^ concentrations were calculated as:

[O_2_^-^] = ((Smp_240nnm_-Smp_400nm_)-(Blk_240nm_-Blk_400nm_))/ 2345M^-1^cm^-1^

Interferences from H_2_O_2_ to the spectrophotometric measurements of superoxide in the illuminated cocktail were assessed as minor as reasoned below. We photo-generated only 1-10 μM O_2_^•-^ and completed the calibration within a maximum of 10 minutes. At this time only 0.25-2.5 μM H_2_O_2_ is expected to be formed from O_2_^•-^ disproportionation and since H_2_O_2_ absorption at 240 nm is 60 fold lower than that of O_2_^•-^ its contribution to the measurement is low. Each calibration line, typically at concentrations between 10-100 nM (Fig. S1) was done by adding small spikes (20-200 μl) of the irradiated cocktail to 50 mL of seawater and following their decay for 20-30 sec. The seawater pH was not affected by these small additions as confirmed by pH measurements at the end of each run. To account for superoxide decay between the spike addition and its detection in the FeLume (typically 20-30 sec, recorded for each spike), the signal was extrapolated to time zero (t_0_) by plotting the natural logarithm of the blank subtracted signal (Fig S1). Higher calibrations slopes were found at lower superoxide concentrations.

*References*

1**. Heyno** E, Klose C, Krieger-Liszkay A **(2008)** Origin of cadmium-induced reactive oxygen species production: mitochondrial electron transfer versus plasma membrane NADPH oxidase. New Phytologist **179**: 687–699

2. O'Donnell BV, Tew DG, Jones OT and England PJ (1993) Studies on the inhibitory mechanism of iodonium compounds with special reference to neutrophil NADPH oxidase. Biochemical Journal **290** (Pt 1): 41-9.

3. Bielski, B. H. J. (1978). "Re-evaluation of spectral and kinetic properties of HO_2_ and O_2_^-^ free radicals." Photochemistry and Photobiology **28**(4-5): 645-649.

4. Koga, S. and M. Nakano (1992). "A high involvement of O2- possibly generated in inner membranes for iron-Induced microsomal lipid-peroxidation." Biochemical and Biophysical Research Communications **186**(2): 1087-1093.
