# Supplementary material for: Extracellular Production and Degradation of Superoxide in the Coral Stylophora pistillata and Cultured Symbiodinium
Source: PLoS One. 2010 Sep 14;5(9):e12508. doi: 10.1371/journal.pone.0012508 (PMC2939047; doi:10.1371/journal.pone.0012508)
Supplement: Table S1 — (0.01 MB DOCX) [file pone.0012508.s002.docx]

**Table S1:** Superoxide antioxidant activity of symbiont (n=4) and aposymbiont (n=5) corals and of cultured *Symbiodinum* from clade C (n=5). The antioxidant activity is expressed as pseudo-first order decay constants normalized to protein, standard deviations are presented in parentheses. The coral antioxidant activity was converted to SOD like activity according to the calibration shown in Fig S4.

|  | **n** | **data type** | **k** | **SOD like activity** |
| --- | --- | --- | --- | --- |
|  |  |  | *(s^-1^ mg^-1^)* | *(U ml^-1^ mg^-1^)* |
| **Non bleached coral** | 4 | range | 0.04 - 0.27 | 0.181 - 1.275 |
|  |  | average | 0.132 (0.106) | 0.614 (0.495) |
| **Bleached coral** | 5 | range | 0.05 - 0.11 | 0.24 - 0.53 |
|  |  | average | 0.088 (0.023) | 0.407 (0.109) |
| **Algae** | 5 | Below detection limit | | |
