# Supplementary figures and images for: Extracellular Production and Degradation of Superoxide in the Coral Stylophora pistillata and Cultured Symbiodinium
Source: PLoS One. 2010 Sep 14;5(9):e12508. doi: 10.1371/journal.pone.0012508 (PMC2939047; doi:10.1371/journal.pone.0012508)

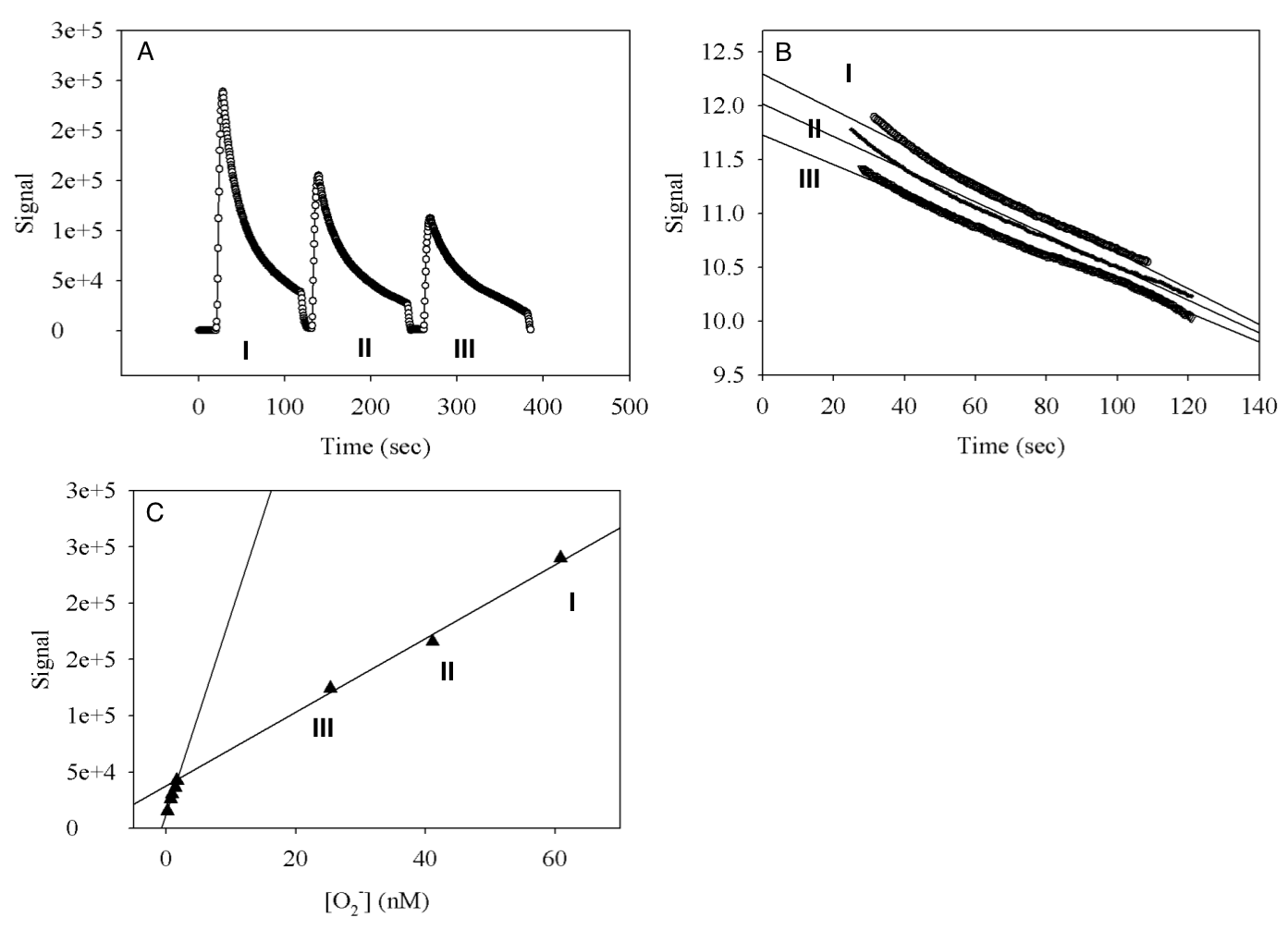

Supplement: Figure S1 — Calibration curve for superoxide in the Felume with MCLA. (A) Superoxide spikes at decreasing concentrations were added to FSW (with 150 µM DTPA) and the decay over time was recorded. (B) The decay curves were plotted in a log linear graph, allowing for back extrapolation to the original signal when the spike was added - t0 (it took ∼30 sec from spike addition to its detection). As expected from pseudo first-order decay kinetics, the O2 − decay curves are linear and their slopes equal to the O2 − decay constant (kFSW). (C). The end product calibration curve of the FeLume MCLA signal at time zero (to) versus the concentrations of O2 − added. All calibration lines were linear (R2 >0.9) for superoxide range used, typically 20–80 nM. However, these lines do not go through the origin, probably because the MCLA response is concentration-dependent over larger ranges. This notion is supported by a second calibration curve conducted with sub nanomolar O2 −, which crosses through the origin and has a higher slope. (4.20 MB TIF) [file pone.0012508.s003.tif]

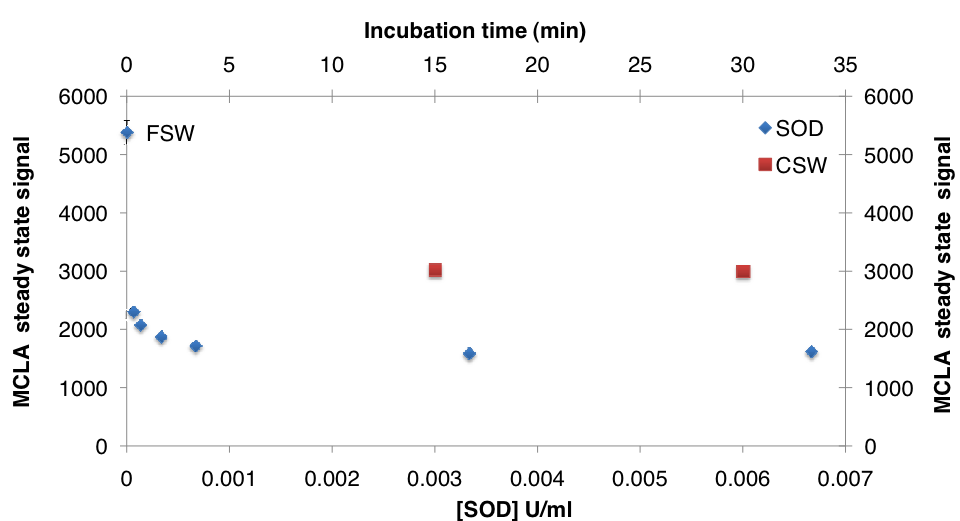

Supplement: Figure S2 — Effect of superoxide dismutase (SOD) on the background signal of MCLA in the presence of superoxide free filtered seawater (FSW, donated by the error). SOD additions even at minimal levels caused a significant quenching of the chemiluminescence signal as was previously reported by Koga and Nakano, 1992 [4], and suggested to result by direct interaction of the enzyme with MCLA or its intermediate derivatives. Filtered seawater incubated with corals (CSW) also had lower background signal compared with FSW, further supporting our hypothesis that the detoxifying agent released from the corals resembles SOD. The detoxifying activity in CSW was converted to SOD units according to the calibration presented in Figure S4. The data is an average of 150 seconds of steady state signal where the standard deviations are too small to be seen. (1.50 MB TIF) [file pone.0012508.s004.tif]

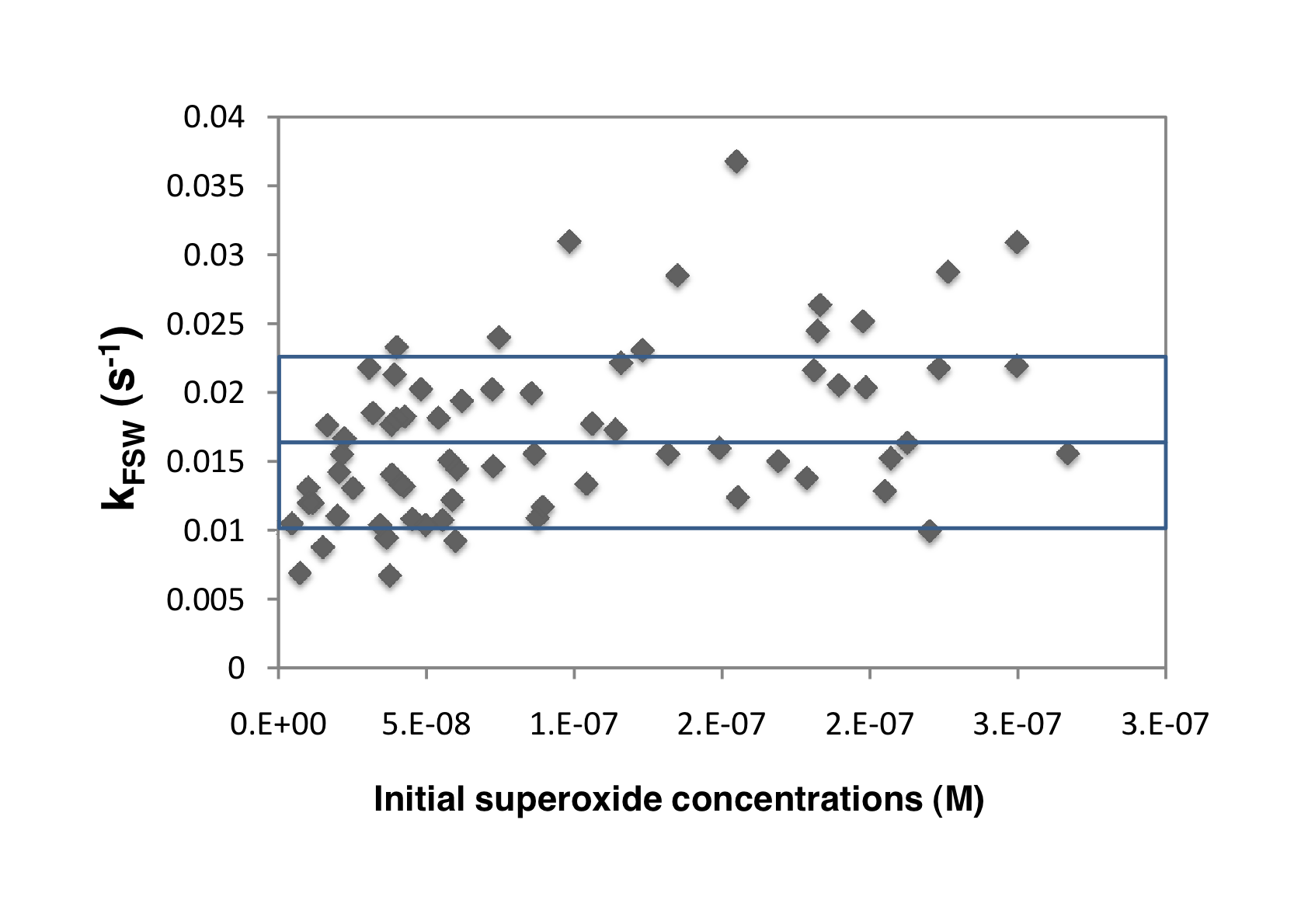

Supplement: Figure S3 — Compilation of all superoxide decay constants (kFSW) in DTPA containing filtered seawater (FSW) obtained with O2 − spikes at different concentrations. Superoxide decay constants were independent of the spike concentrations (R2 = 0.199), indicating that the reaction is pseudo first-order and reaffirming our data analysis approach (Fig. S1). The dotted line represents the average superoxide decay constant in FSW, which serves as a background for the coral induced elevated O2 − decays. The grey area represents 1 standard deviation (1SD) on the average. (6.01 MB TIF) [file pone.0012508.s005.tif]

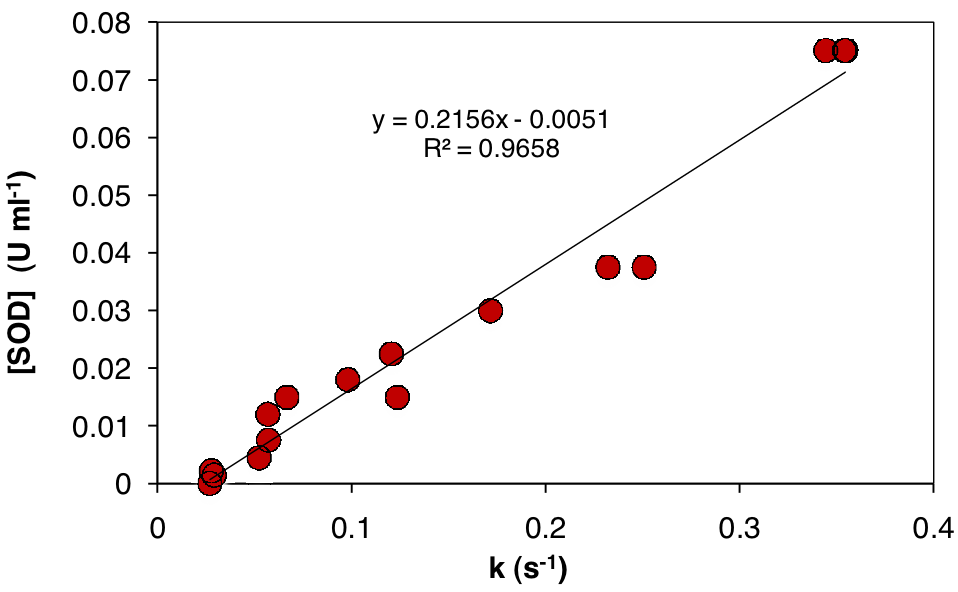

Supplement: Figure S4 — Calibration between the activity of commercial SOD and superoxide decay rates (expressed as pseudo first order decay constant k). Having established that the coral antioxidant activity resembles that of SOD (Figure 5), this curve enables the conversion of the experimentally measured superoxide decay rates to SOD activity, as done in Figure S2. (1.70 MB TIF) [file pone.0012508.s006.tif]
